# Preclinical PET imaging of glycoprotein non-metastatic melanoma B in triple negative breast cancer: feasibility of an antibody-based companion diagnostic agent

**DOI:** 10.18632/oncotarget.22228

**Published:** 2017-11-01

**Authors:** Bernadette V. Marquez-Nostra, Supum Lee, Richard Laforest, Laura Vitale, Xingyu Nie, Krzysztof Hyrc, Tibor Keler, Thomas Hawthorne, Jeremy Hoog, Shunqiang Li, Farrokh Dehdashti, Cynthia X. Ma, Suzanne E. Lapi

**Affiliations:** ^1^ Mallinckrodt Institute of Radiology, Washington University School of Medicine, St. Louis, MO, USA; ^2^ Department of Radiology, University of Alabama at Birmingham, Birmingham, AL, USA; ^3^ Department of Radiology and Biomedical Imaging, PET Center, Yale University School of Medicine, New Haven, CT, USA; ^4^ Celldex Therapeutics, Hampton, NJ, USA; ^5^ The Hope Center for Neurological Disorders, Washington University School of Medicine, St. Louis, MO, USA; ^6^ Department of Medicine, Washington University School of Medicine, St. Louis, MO, USA

**Keywords:** PET imaging, triple negative breast cancer, glycoprotein non-metastatic melanoma B, dosimetry, glembatumumab

## Abstract

High levels of expression of glycoprotein non-metastatic B (gpNMB) in triple negative breast cancer (TNBC) and its association with metastasis and recurrence make it an attractive target for therapy with the antibody drug conjugate, glembatumumab vedotin (CDX-011). This report describes the development of a companion PET-based diagnostic imaging agent using ^89^Zr-labeled glembatumumab ([^89^Zr]DFO-CR011) to potentially aid in the selection of patients most likely to respond to targeted treatment with CDX-011. [^89^Zr]DFO-CR011 was characterized for its pharmacologic properties in TNBC cell lines. Preclinical studies determined that [^89^Zr]DFO-CR011 binds specifically to gpNMB with high affinity (Kd = 25 ± 5 nM), immunoreactivity of 2.2-fold less than the native CR011, and its cellular uptake correlates with gpNMB expression (r = 0.95). In PET studies at the optimal imaging timepoint of 7 days p.i., the [^89^Zr]DFO-CR011 tumor uptake in gpNMB-expressing MDA-MB-468 xenografts had a mean SUV of 2.9, while significantly lower in gpNMB-negative MDA-MB-231 tumors with a mean SUV of 1.9. [^89^Zr]DFO-CR011 was also evaluated in patient-derived xenograft models of TNBC, where tumor uptake *in vivo* had a positive correlation with total gpNMB protein expression via ELISA (r = 0.79), despite the heterogeneity of gpNMB expression within the same group of PDX mice. Lastly, the radiation dosimetry calculated from biodistribution studies in MDA-MB-468 xenografts determined the effective dose for human use would be 0.54 mSv/MBq. Overall, these studies demonstrate that [^89^Zr]DFO-CR011 is a potential companion diagnostic imaging agent for CDX-011 which targets gpNMB, an emerging biomarker for TNBC.

## INTRODUCTION

The rise of companion diagnostic imaging agents, which are molecular imaging agents that provide information on the effective use of the corresponding drugs, is changing the paradigm of screening for targeted therapy in cancer. Companion diagnostics typically make use of *in vitro* testing of biopsied tissues using immunohistochemistry, fluorescence *in situ* hybridization, enzyme-linked immunosorbent assays, quantitative PCR, or DNA sequencing, among others [1]. While important for diagnosis, these techniques do not provide information on the location of therapeutic targets and the delivery of the therapeutic agents throughout the body. Nuclear imaging of the expression of therapeutic targets has the potential to identify patients who may benefit from such therapies while sparing patients unlikely to respond to unnecessary treatments. Several examples have demonstrated the potential of PET imaging using ^89^Zr-labeled antibodies as companion diagnostic agents to (i) assess receptor targets *in vivo* especially in the metastatic setting, (ii) identify candidate patients for targeted therapy, and (iii) determine the overall safety of these imaging agents [2–4]. In breast cancer, [^89^Zr]DFO-trastuzumab is paving the way towards the selection of the human epidermal growth factor receptor 2 (HER2)-positive patients for therapeutic antibodies that target this receptor. While this companion diagnostic imaging agent shows promise in stratifying patients who may benefit from the already established HER2-targeted therapies, there is currently no such approach for patients with the triple negative breast cancer (TNBC) subtype.

TNBC is diagnosed by the absence of three receptors that can be targeted for therapy: HER2, estrogen receptor, and progesterone receptor. The current standard of care for patients diagnosed with TNBC is chemotherapy, but resistance to these treatments and early recurrence of disease are prevalent [5, 6]. TNBC patients are also more likely to develop metastases in the brain, bone, lung, and liver [7]. For these reasons, TNBC is more aggressive than other subtypes of breast cancer. However, targeted treatments in clinical trials are currently gaining traction to improve patient outcomes such as immune therapy and antibody drug conjugates (ADCs) [8, 9]. In general, ADCs deliver the drug payload specifically to cancer cells by binding to receptors that are overexpressed on the cancer cell surface, are internalized by the cell, and release the drug to kill the cell via different mechanisms specific for each type of ADC. ADCs have shown success in the treatment of the HER2-positive breast cancer subtype [10, 11].

Using similar strategies to existing companion diagnostic imaging agents for the HER2-positive breast cancer subtype, we have developed a positron emission tomography (PET) agent for imaging gpNMB. This transmembrane glycoprotein is overexpressed in many malignancies [12–14]. gpNMB is expressed in ≥ 25% of tumor epithelial cells in about 40% of TNBC patients [9]. In most normal cells, gpNMB is expressed intracellularly, which permits greater selectivity for targeting malignant cells via the extracellular domain of this protein [15]. gpNMB is most commonly expressed in the basal-like subtype of TNBC and has been found to promote metastasis and is linked to recurrence [12, 13]. Higher levels of gpNMB expression in TNBC are associated with worse metastasis-free survival and overall survival (OS). Preclinical studies in melanoma have shown that the level of gpNMB expression is proportional to the effectiveness of gpNMB-targeted therapy via an ADC called glembatumumab vedotin (CDX-011) [16, 17].

Previous clinical trials have demonstrated an acceptable safety profile of CDX-011 and improved progression-free survival (PFS) of patients with gpNMB-positive breast cancer regardless of their subtype [9, 18]. In these studies, the greatest impact (objective response rate (ORR), PFS, and OS) of CDX-011 relative to the control arm was observed in patients with TNBC that expressed gpNMB. In the EMERGE trial, increasing levels of gpNMB expression (≥ 5% vs ≥ 25% positivity) are associated with higher response rates (15% vs 30%, respectively) to CDX-011 [9]. The efficacy of CDX-011 is being compared to capecitabine in the ongoing phase II METRIC trial in patients with TNBC positive for gpNMB in >25% of tumor epithelium by IHC of an archival tumor material (NCT#01997333)[19]. Imaging gpNMB will provide a non-invasive means for identifying patients who may benefit from treatment with CDX-011. Taken together, the expression of gpNMB plays an important role in clinical outcome, making gpNMB an attractive target for imaging and therapy of TNBC.

In this report, we describe the development of a companion diagnostic imaging agent to image gpNMB in xenograft models of TNBC. Our approach is to radiolabel the naked antibody, glembatumumab (CR011), with a PET radionuclide, Zr-89 (t_1/2_ = 3.3 days). The sensitivity of PET combined with the decay properties of Zr-89 could be advantageous in providing high resolution images and quantitative value in tracking the distribution of the antibody *in vivo* well into its clearance from the bloodstream [16]. Therefore, [^89^Zr]DFO-CR011 will provide a non-invasive, quantitative, and sensitive assessment of gpNMB expression in preclinical models of TNBC and eventually in clinical trials. In this work, we show the development of [^89^Zr]DFO-CR011 as a companion diagnostic imaging agent via preclinical evaluation to pre-qualify this PET agent for translation to clinical trial. Toward this end, we evaluated its specificity for gpNMB, immunoreactivity, stability, imaging quality, pharmacokinetic properties, and dosimetry; all of which are required for an investigational new drug (IND) application to the FDA.

## RESULTS

### Characterization of [^89^Zr]DFO-CR011 *in vitro*

The ratio of DFO:CR011 was determined to be 4 (Supplementary Figure 1), which is comparable to that of the drug:antibody ratio of 4 - 5 in CDX-011 [20–22]. The stability of [^89^Zr]DFO-CR011 was determined in buffer at ambient temperature for 24 hrs, as this time point under these conditions could be used to set an expiration time for injection into patients from the end of antibody radiolabeling. FPLC analysis showed the stability of [^89^Zr]DFO-CR011 with a retention time of 22.5 min, radiochemical purity of 100%, and antibody aggregation of 7% at t_0_ and 10% at 24 hrs (Supplementary Figure 2). This aggregation meets the specification of up to 20% aggregation based on the criterion used for the chemistry, manufacturing, and controls of an IND-approved [^89^Zr]-trastuzumab, which means that an expiration time of 24 hrs can be used for injection into patients [23]. Further, a serum stability study was performed in separate solutions of human and mouse sera up to 7 days, *in vitro*. We found that [^89^Zr]DFO-CR011 is more stable in human serum than in mouse serum. In human serum, [^89^Zr]DFO-CR011 was 97% monomer initially, was maintained at 95% monomer at 5 days, and decreased to 67% at 7 days. In mouse serum, [^89^Zr]DFO-CR011 was 97% monomer initially, and decreased to 64, 59, and 57 % at 1, 5, and 7 days, respectively (Supplementary Figure 3).

[^89^Zr]DFO-CR011 was then characterized using various cell binding assays *in vitro*. Human TNBC cell lines were confirmed for cell surface expression of gpNMB using flow cytometry (Supplementary Figure 4) and quantified total gpNMB expression from whole cell lysate using ELISA (Supplementary Table 1). MDA-MB-157, MDA-MB-468, and MDA-MB-231 were determined to have high (11.1 ± 0.9 pg gpNMB/μg total protein), moderate (2.7 ± 0.1), and negligible (0.15 ± 0.05) expression of gpNMB, respectively (Supplementary Table 1), which is in agreement with the flow cytometry results (Supplementary Figure 4). As a control, we used the SK-Mel2 human melanoma cells, as this cell line highly expresses gpNMB (6.8 ± 0.3) [17].

**Figure 1:**
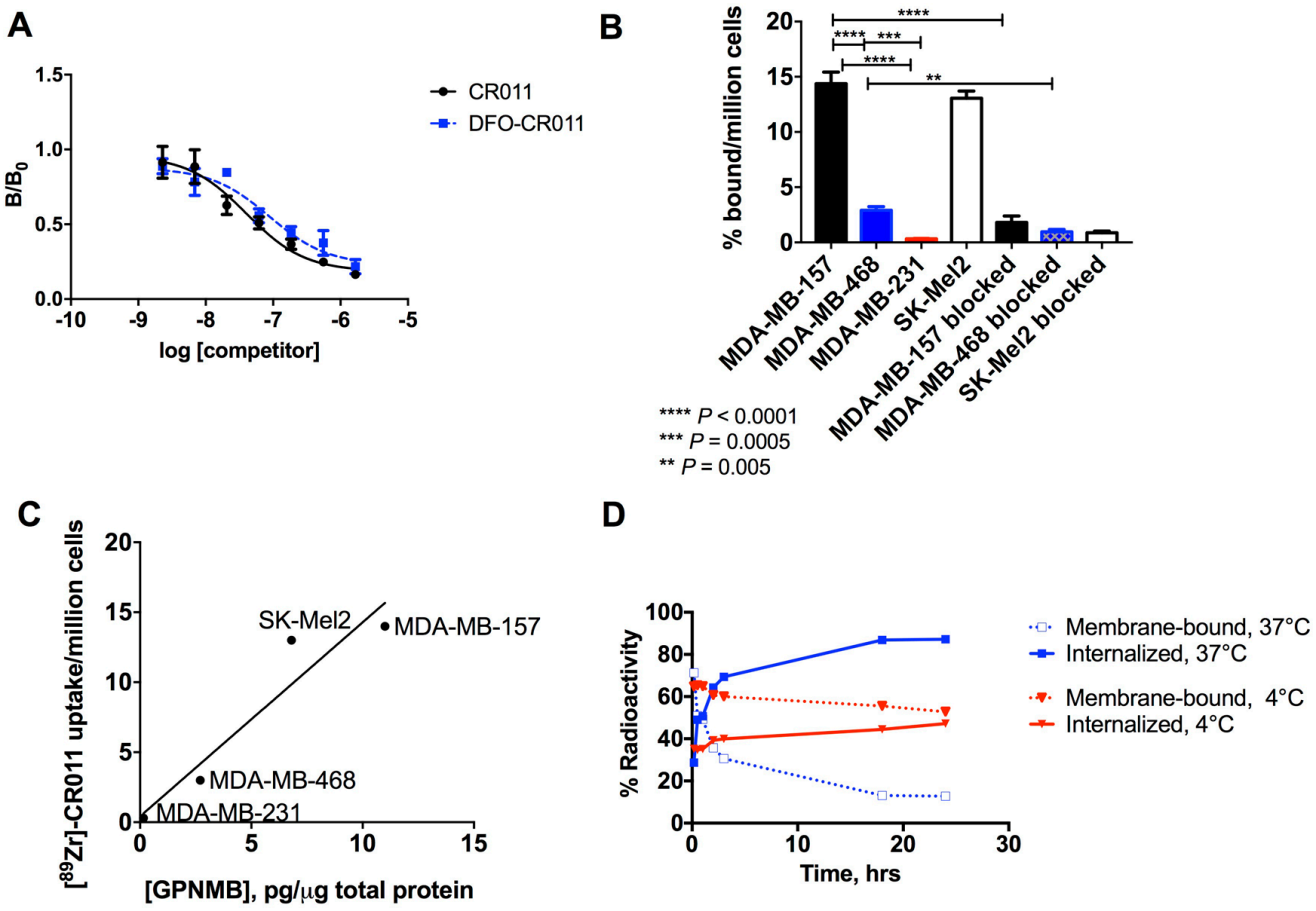
Pharmacologic properties of [^89^Zr]DFO-CR011 *in vitro* **(A)** Competitive binding study in MDA-MB-468. **(B)** Cell uptake and blocking studies in cell lines with varying gpNMB expression. **(C)** The cell uptake of [^89^Zr]DFO-CR011 is correlated with gpNMB expression in whole cell lysates (Pearson’s r = 0.95). **(D)** Rate of cellular internalization at 37°C showed 50% of bound [^89^Zr]DFO-CR011 internalized within 30 – 60 min. The reaction at 4°C significantly reduced cellular internalization.

The immunoreactivity was determined using a competitive binding assay, where the DFO-CR011 conjugate retained its antigen-binding property with a 2.2-fold ratio between EC_50_ values for DFO-CR011 (EC_50_ = 88 nM) and native CR011 (EC_50_ = 40 nM) (Figure 1A). Additionally, the binding affinity (Kd) of [^89^Zr]DFO-CR011 and copy number of gpNMB (Bmax) were determined via a radioligand saturation assay in SK-Mel2, MDA-MB-157, and MDA-MB-468, resulting in an average Kd of 25 ± 5 nM in these cell lines (Supplementary Figure 5A). This Kd value is in agreement with previous studies reporting a Kd value of 52 nM for native CR011 against recombinant gpNMB using non-radioactive methods [20]. Based on these saturation assays, we interpolated Bmax values to determine the number of gpNMB molecules per cell. We found that there are about 605 ± 28 k gpNMB per MDA-MB-157 cell; 246 ± 5 k in MDA-MB-468; and 637 ± 12 k in SK-Mel2 (Supplementary Figure 5). Further, cell uptake studies showed [^89^Zr]DFO-CR011 bound specifically to MDA-MB-157 and MDA-MB-468 cells at 2 hrs of incubation with 14 ± 1 and 3 ± 0.3 % bound/million cells, respectively, and can delineate different expression levels of gpNMB (Figure 1B). The high uptake in MDA-MB-157 was similar to that of the SK-Mel2 positive control with 13 ± 0.6 % bound/million cells. Blocking with excess unlabeled CR011 significantly reduced [^89^Zr]DFO-CR011 uptake in MDA-MB-157, MDA-MB-468, and SK-Mel2 to 2 ± 0.6, 1 ± 0.2, and 0.8 ± 0.1 % bound/million cells, respectively. As expected, the uptake in gpNMB-negative MDA-MB-231 cells was minimal with 0.3 ± 0.03 % bound/million cells. The cell uptake of [^89^Zr]DFO-CR011 in these cell lines was correlated with gpNMB expression on whole cell lysates, as determined via ELISA. The Pearson’s correlation coefficient was determined to be 0.95 (Figure 1C).

As the effectiveness of antibody drug conjugates rely on the internalization of the antibody to the cell to deliver the drug, we evaluated the rate of cellular internalization of [^89^Zr]DFO-CR011 in MDA-MB-468 cells. We found that 50% of bound [^89^Zr]DFO-CR011 was internalized within 30 – 60 min at 37°C, whereas [^89^Zr]DFO-CR011 did not reach 50% internalization within the 24-hr time point at 4°C (Figure 1D). Taken together, these results show the feasibility of [^89^Zr]DFO-CR011 as an excellent imaging agent for gpNMB. Its specificity, high affinity, and rapid cellular internalization indicate that [^89^Zr]DFO-CR011 could be an excellent companion diagnostic agent for CDX-011 and a PET imaging agent for gpNMB *in vivo*.

### [^89^Zr]DFO-CR011 is specific to gpNMB in cell-derived xenograft models of TNBC

**Figure 2:**
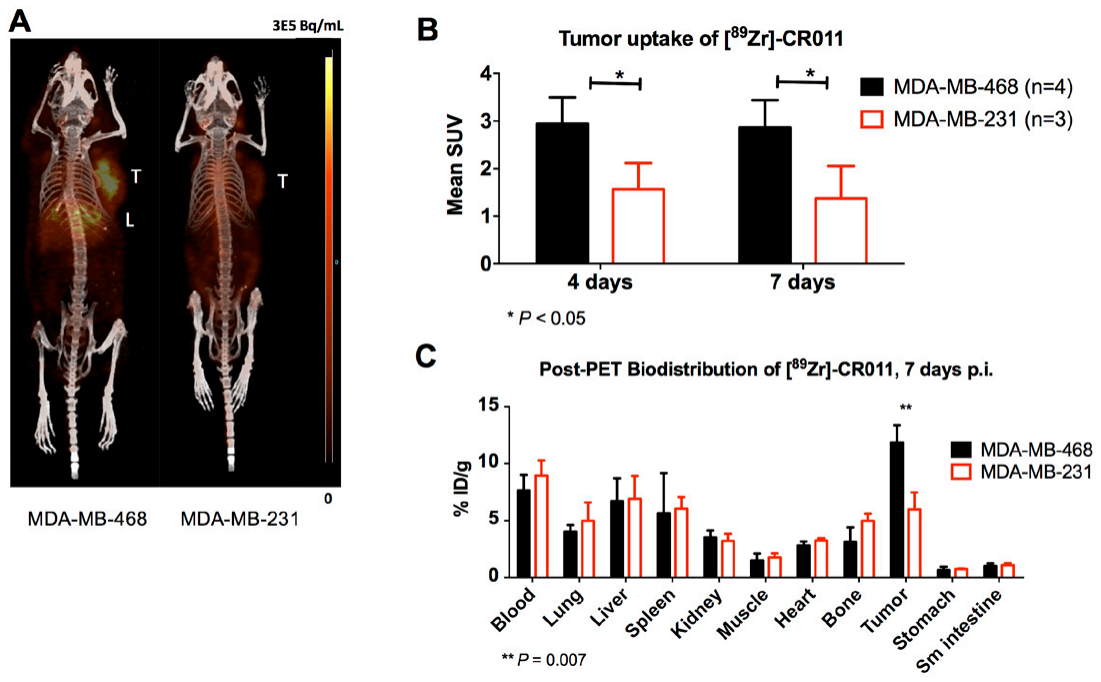
PET/CT imaging of [^89^Zr]DFO-CR011 in TNBC xenograft models at 4 days post-injection **(A)** Maximum Intensity Projections of representative mice. **(B)** Mean SUV in tumors at 4 and 7 days show significantly higher [^89^Zr]DFO-CR011 uptake in MDA-MB-468 at both time points. **(C)** Biodistribution study post PET imaging at 7 days. Similar [^89^Zr]DFO-CR011 uptake in normal organs were observed for both xenograft models.

The pharmacokinetic properties were determined via biodistribution studies at 1, 4, 7, and 12 days post injection (p.i.) in MDA-MB-468 xenografts, where the tumor uptake of [^89^Zr]DFO-CR011 increased from 13.3 ± 1.8 % ID/g at 1 day p.i. to 22.1 ± 2.0 % ID/g at 4 days p.i. (Table 1). The tumor uptake was constant at 7 and 12 days p.i., with 24.5 ± 7.7 and 23.5 ± 2.9 % ID/g, respectively, confirming the residualizing property of Zr-89. The uptake in the blood, heart, and liver also decreased, showing the slow clearance or metabolism of [^89^Zr]DFO-CR011 over time. Metabolism studies are needed for future investigations to elucidate the cause of decreased uptake of this radiotracer in these organs over time. The biodistribution of this fully human [^89^Zr]DFO-CR011 in normal organs of mice was consistent with that of other ^89^Zr-labeled antibodies that are specific for their human antigens [23, 24]. As expected, radioactive uptake in the bone had a steady increase over the 12 days from 2.4 ± 0.2 %ID/g at 1 day p.i. to 10.3 ± 2.1 %ID/g at 12 days p.i., which is likely due to the decomplexation of Zr-89 from the antibody-DFO conjugate (Table 1) [25]. Additionally, the specificity of [^89^Zr]DFO-CR011 was confirmed under blocking conditions from pre-administration of 1 mg of unlabeled CR011 at 1 day prior to the injection of [^89^Zr]DFO-CR011 in a subset of mice. The tumor uptake of [^89^Zr]DFO-CR011 was reduced by 50% at 4 days p.i. (Table 1). Based on these studies, we performed PET imaging at 4 and 7 days p.i., where optimal tumor-to-background (T/B) ratios were observed in addition to maintaining imaging quality. PET imaging of [^89^Zr]DFO-CR011 in MDA-MB-468 was compared to that in the gpNMB-negative MDA-MB-231 xenograft. Figure 2A shows maximum intensity projections of MDA-MB-468 and MDA-MB-231, with higher tumor uptake in the gpNMB-positive xenograft. Quantification of tumor uptake at 4 and 7 days p.i. determined that [^89^Zr]DFO-CR011 had a mean SUV of 2.9 ± 0.6 in the gpNMB-positive xenograft versus 1.6 ± 0.5 in the gpNMB-negative model (P < 0.05) at 4 days p.i. (Figure 2B). The same difference in uptake was observed at 7 days p.i. with a mean of 2.9 ± 0.5 for MDA-MB-468, versus 1.4 ± 0.7 for MDA-MB-231 (P < 0.05). A post-PET biodistribution study was performed to compare [^89^Zr]DFO-CR011 uptake in normal organs and validate quantification from imaging studies. No differences in organ uptake were observed for these different models, with the exception of the tumor (Figure 2C).

**Table 1:** Biodistribution of [^89^Zr]DFO-CR011 in MDA-MB-468 xenografts under non-blocking and blocking conditions from 1 – 12 days post injection (n = 3 per group)

Organ	1 day p.i. (%ID/g ± SD)			1 day p.i. blocking (%ID/g ± SD)			4 days p.i. (%ID/g ± SD)			4 days p.i. blocking (%ID/g ± SD)			7 days p.i. (%ID/g ± SD)			12 days p.i. (%ID/g ± SD)		
Blood	23.4	±	2.8	22.6	±	1.6	17.7	±	4.8	14.7	±	1.9	13.1	±	1.2	9.2	±	0.6
Lung	9.7	±	0.6	10.6	±	1.6	8.6	±	3.0	7.4	±	1.3	7.0	±	0.9	5.8	±	0.7
Liver	12.7	±	6.1	7.1	±	2.8	9.6	±	3.7	5.2	±	0.9	6.7	±	0.9	7.4	±	3.3
Spleen	5.6	±	1.0	6.9	±	1.1	7.8	±	3.4	6.5	±	2.2	7.4	±	1.3	7.7	±	1.5
Kidney	5.6	±	0.6	5.7	±	1.0	5.1	±	1.4	4.5	±	0.7	4.5	±	0.5	4.5	±	0.2
Muscle	3.0	±	0.6	2.9	±	0.3	2.3	±	0.7	2.2	±	0.3	2.0	±	0.4	1.7	±	0.1
Heart	8.3	±	2.4	6.9	±	0.9	5.9	±	2.3	4.6	±	1.1	5.1	±	0.5	2.9	±	0.1
Bone	2.4	±	0.2	2.2	±	0.3	5.4	±	1.9	4.3	±	0.6	6.7	±	0.8	10.3	±	2.1
Marrow	7.8	±	0.6	7.5	±	0.5	6.9	±	1.9	5.5	±	1.4	6.4	±	2.1	5.9	±	0.6
Tumor	13.3	±	1.9	11.0	±	2.2	22.1	±	2.0	10.0	±	2.0	24.5	±	7.7	23.5	±	2.9
Stomach	1.0	±	0.3	1.0	±	0.2	0.8	±	0.2	0.8	±	0.5	1.3	±	0.8	0.7	±	0.2

Values represent percent injected dose per gram ± standard deviation (%ID/g ± SD).

**Figure 3:**
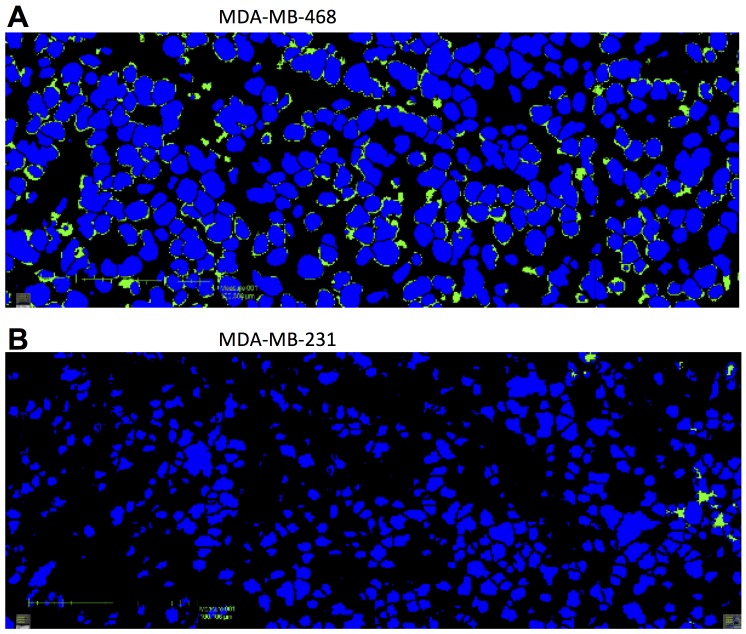
Immunofluorescence staining of gpNMB in MDA-MB-468 **(A)** and MDA-MB-231 **(B)** tumor sections. Scale bars represent 50 μm. DAPI stained nuclei are marked blue and gpNMB stained (positive) membrane fragments are shown as green.

Interestingly, the tumor uptake in MDA-MB-468 from the biodistribution study following PET (post-PET biodistribution) was about 50 % lower than that from the biodistribution in the pharmacokinetic study at 7 days p.i., with 11.9 ± 1.5 and 24.5 ± 7.7 % ID/g, respectively. In the post-PET biodistribution study, we injected 20 μg (3.7 MBq) of [^89^Zr]DFO-CR011 in order to obtain a high imaging signal, compared with 10 μg (1.9 MBq) of [^89^Zr]DFO-CR011 for the pharmacokinetic study. As the specific activity is equivalent in both studies (185 MBq/mg), the 20 μg-injected dose in the post-PET biodistribution study likely saturated gpNMB and, thus, yielded a lower % ID/g at 7 days p.i. To test this hypothesis, we then conducted a biodistribution study in a small subset of mice injected with 2 μg (0.37 MBq) of [^89^Zr]DFO-CR011 at 7 days p.i. to determine if a lower mass of antibody would increase tumor uptake. We found no significant increase in the tumor uptake for the 2 μg dose (20.4 ± 4.6 % ID/g) compared with the 10 μg dose (Supplementary Figure 6). Rather, we observed a trend of increasing tumor:blood ratios with decreasing mass of antibody. Tumor:blood ratios determined from biodistribution studies at 7 days p.i. were 1.55 ± 0.05, 1.86 ± 0.22, and 1.92 ± 0.14 which correspond to the 20, 10, and 2 μg of [^89^Zr]DFO-CR011, respectively. Tumor:blood ratios for the 20 and 2 μg doses were statistically significant (*P* = 0.03), which could be due to a blocking effect. However, other dose comparisons were not statistically significant (*P* > 0.05), and could be due to shed antigen. While CR011 would be able to bind to shed gpNMB, we did not test for the presence of this shed antigen in our animal models due to limited volume of blood samples. More investigations are needed to determine the role of specific activity, mass of antibody, and quantification of shed antigen in the translation of [^89^Zr]DFO-CR011 to the clinical setting in order to optimize the sensitivity of detecting gpNMB. Nevertheless, [^89^Zr]DFO-CR011 can differentiate low expression of gpNMB as compared with negative expression *in vivo* (Figure 2B). Immunofluorescence staining of gpNMB on these tumors *ex vivo* is consistent with the imaging and biodistribution studies (Figure 3). Raw data and H&E stains are shown in Supplementary Figure 7. Despite the weak intensity of extracellular gpNMB staining in the MDA-MB-468, PET using [^89^Zr]DFO-CR011 was capable of detecting gpNMB *in vivo*.

### Dosimetry of [^89^Zr]DFO-CR011 in MDA-MB-468 xenografts

Data from biodistribution studies were used to determine radiation dosimetry. Organ residence times are presented in Table 2. Due to the long circulation time of antibodies, the blood residence times are observed to be the highest with 23.6 hrs. The decay-corrected uptake in the blood over time shows that the effective half-life of CR011 was about 8 days (Supplementary Figure 8A), which is consistent with previous studies reporting a 10-day half-life using non-radioactive methods [20]. Blood-rich organs such as liver and lungs also depict long residence times. The rate of tumor uptake was also determined to be 0.034% ID/g/hr, which corresponds to a half-life of 20 hrs (Supplementary Figure 8B). However, the long blood residence time limits imaging at this earlier time point. The organ and whole body radiation dosimetry is presented in Table 3. The biodistribution, typical of antibodies, and the long tracer retention time leads to an approximately 0.54 mSv/MBq dose in each organ. The heart appears to be the dose-limiting organ due to the long retention in the blood. A high radiation dose is also observed in the osteogenic cells. The effective dose is measured at 0.54 mSv/MBq. Under NRC CFR 361.1 regulation, these values indicate a maximum recommended dose of 90 MBq (2.2 mCi) would be feasible for patient studies.

**Table 2:** Residence times of [^89^Zr]DFO-CR011 in MDA-MB-468 xenografts

Organ	Residence time, hrs
Blood	23.6 ± 3.4
Lungs	2.65 ± 0.41
Liver	5.54 ± 2.04
Spleen	0.45 ± 0.10
Kidneys	0.56 ± 0.07
Muscle	16.3 ± 3.3
Fat	9.4 ± 3.2
Heart	0.66 ± 0.15
Bone	7.68 ± 1.59
Red Marrow	3.61 ± 0.62
Pancreas	0.08 ± 0.01
Stomach	0.06 ± 0.02
Small Intestine	0.39 ± 0.07
Upper L. Intestine	0.11 ± 0.02
Lower L. Intestine	0.08 ± 0.02
Heart Content	2.36 ± 0.34
Remainder of the body	72.5

**Table 3:** Predicted organ radiation doses for humans

Organ	(mSv/MBq)
Adrenals	0.67
Brain	0.49
Breasts	0.45
Gallbladder Wall	0.65
LL Intestine Wall	0.62
Small Intestine Wall	0.57
Stomach Wall	0.56
UL Intestine Wall	0.62
Heart Muscle	1.04
Kidneys	0.63
Liver	0.92
Lungs	0.71
Muscle	0.47
Ovaries	0.61
Pancreas	0.62
Red Marrow	0.63
Osteogenic Cells	1.04
Skin	0.37
Spleen	0.71
Thymus	0.60
Thyroid	0.49
Urinary	0.45
Uterus	0.62
Total	0.55
Effective Dose	0.54

### Evaluation of [^89^Zr]DFO-CR011 in patient-derived xenograft models of TNBC

**Figure 4:**
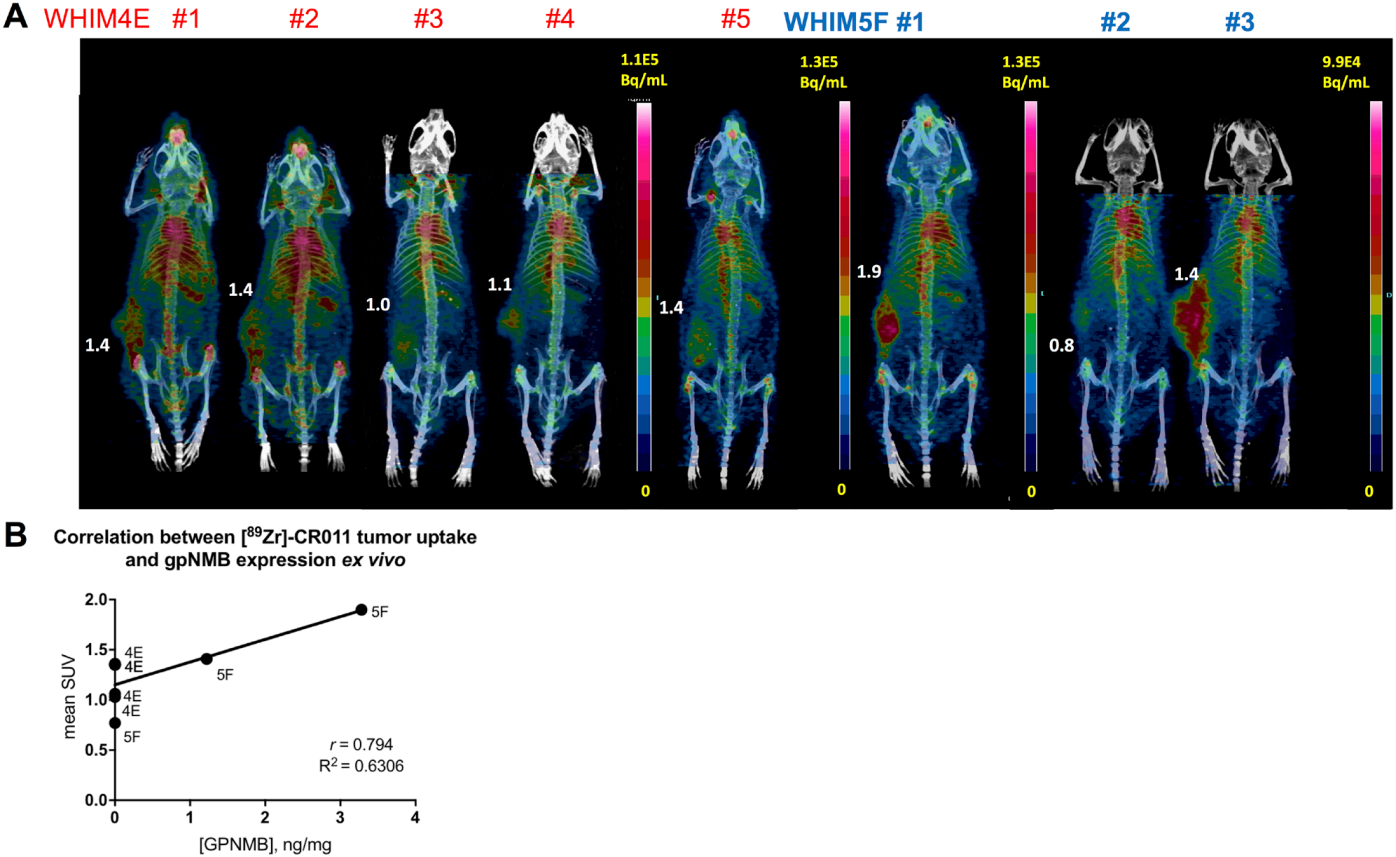
PET/CT imaging of [^89^Zr]DFO-CR011 in PDX models of TNBC at 7 days post-injection **(A)** Maximum Intensity Projections of WHIM-4E and WHIM-5F. Mean SUV are shown as values next to the tumors. **(B)** Correlative study between mean SUV in the tumor and gpNMB expression via ELISA.

A panel of PDX breast tumor tissues were screened for mRNA expression of gpNMB at different passages (Supplementary Figure 9). WHIM-4 and WHIM-5 models were selected for imaging studies, as these PDX models expressed high and low mRNA levels of gpNMB, respectively (Supplementary Figure 9). Immunohistochemistry confirmed the relative protein expression of gpNMB in earlier passages (A – D) of these models that were available (Data not shown). PET using [^89^Zr]DFO-CR011 was performed in passage E of WHIM-4 and F of WHIM-5 at 4 and 7 days p.i., similarly to imaging of MDA-MB-468 and MDA-MB-231 xenograft models (Figure 4A). Total protein expression of gpNMB was then confirmed following PET studies using ELISA and IHC. This study shows that gpNMB expression was heterogeneous in WHIM5F (n = 3) and lost expression in WHIM4E (n = 5) (Figure 4A). Mean SUV of the [^89^Zr]DFO-CR011 tumor uptake was correlated with total gpNMB expression obtained from ELISA (Figure 4B). Supplementary Figure 10 confirms the membrane and cytoplasmic staining of gpNMB although quantification using this technique was not performed due to the heterogeneous expression of gpNMB within each PDX tumor sample. Thus, we simplified our correlative analysis to total gpNMB expression using ELISA. Despite the heterogeneity of gpNMB expression, a positive correlation was determined between [^89^Zr]DFO-CR011 tumor uptake *in vivo* and total gpNMB expression *ex vivo* (r = 0.79, Figure 4B).

## DISCUSSION

There is an unmet need to validate novel targets for TNBC. The gpNMB is an example of an emerging biomarker which is overexpressed in up to 40% of TNBC. For this reason, the anti-gpNMB antibody drug conjugate, CDX-011, has been accelerated into clinical trials for the treatment of patients with advanced TNBC, notably those who are refractory to standard of care chemotherapy and demonstrated efficacy in gpNMB over expressing tumors [9]. Incorporation of companion diagnostic imaging agents in clinical trials of CDX-011 could assist the selection of patient candidates. Currently, gpNMB expression is determined by IHC for patient eligibility. While IHC is a valuable tool, a significant limitation is the sampling of a small piece of the tumor often from an archival specimen which may not be accurately representative of the patients’ tumor lesion or distant lesions. In advanced TNBC, the heterogeneity of gpNMB expression in metastatic lesions is currently unknown. Further, IHC cannot delineate membrane from cytoplasmic gpNMB expression. PET imaging using [^89^Zr]DFO-CR011 could provide information about the heterogeneity of gpNMB expression and quantify cell surface gpNMB, a requirement for CDX-011 therapy. While tissue distribution of the radiolabeled naked antibody may not be exactly the same as that of the ADC, the affinity for gpNMB is similar and is currently the best approach for a companion diagnostic imaging agent despite this inherent limitation. Additionally, shedding of gpNMB extracellular domain via the ADAM10 sheddase activity [26] might potentially render CDX-011 therapy less effective. PET imaging using [^89^Zr]DFO-CR011 could potentially predict the effectiveness of CDX-011.

In this study, we sought to evaluate [^89^Zr]DFO-CR011 in preclinical models of TNBC to determine the feasibility of this radiopharmaceutical for translation to clinical trial as a companion diagnostic agent to CDX-011. We chose Zr-89 over other radionuclides due to its favorable half-life (3.3 days) that matches the 8-day biological half-life of CR011 in mice (Supplementary Figure 6B) to allow for imaging at later time points. Its desired decay properties also permit high resolution imaging, and its residualizing property could minimize the translocation of Zr-89 to other organs after cellular internalization. Leveraging established chemistries and the success of CDX-011 in Phase I/II clinical trials, we applied [^89^Zr]DFO-CR011 PET in triple negative breast cancer which currently lacks established targeted treatments. Our work can serve as the basis to translate [^89^Zr]DFO-CR011 to the clinic as a companion diagnostic agent to CDX-011 with the potential to accelerate its FDA-approval by providing a means to identify patients who might benefit from this treatment strategy. Our results show that [^89^Zr]DFO-CR011 binds specifically to gpNMB and can distinguish different levels of cell surface gpNMB expression based on our cell binding studies and imaging *in vivo* (Table 1, Figures 1, 2, and 4). Despite the heterogeneity observed in the clinically-relevant PDX models, the sensitivity of detecting gpNMB in these experiments and in the more established cell-derived xenograft models shows that [^89^Zr]DFO-CR011 shows promise in delineating the varying expression levels of gpNMB in patients whose ability to respond to CDX-011 is dependent on the level of gpNMB expression [9]. Thus, PET using [^89^Zr]DFO-CR011 has the potential to predict response to CDX-011 in future investigations.

However, a limitation of this study is that we were unable to assess the selectivity of [^89^Zr]DFO-CR011 for targeting gpNMB in the tumor versus gpNMB in normal organs, as the CR011 antibody binds selectively to human gpNMB. Despite this limitation, a dosimetry study in mice is still required for clinical translation to determine an estimated dose for human use (Table 3). PET imaging in humans would be a more accurate way to assess the selectivity of [^89^Zr]DFO-CR011 for gpNMB-overexpressing tumors over normal organs. Thus, this concept warrants further investigation in a clinical setting. Toward this goal, we prepared [^89^Zr]DFO-CR011 similarly to other ^89^Zr-labeled antibodies currently approved for clinical investigations [23, 27] to estimate the radiation dosimetry for human use, which is consistent with existing ^89^Zr-labeled antibodies that are currently being investigated in clinical trials [23].

In conclusion, this work presents the first time that gpNMB is imaged *in vivo*. PET imaging of gpNMB using [^89^Zr]DFO-CR011 in these preclinical studies demonstrates the feasibility of translating this radiopharmaceutical to a clinical trial to select patients with TNBC who might benefit from CDX-011. Further, imaging gpNMB in the metastatic setting will determine its overall expression throughout the body, and assess if heterogeneity of gpNMB plays a role in patient outcome. Lastly, PET imaging of gpNMB could be applied to other cancer types that overexpress this clinically-relevant target beyond triple negative breast cancer.

## MATERIALS AND METHODS

### Cell lines and animal models

The human TNBC cell lines (MDA-MB-157, MDA-MB-468 and MDA-MB-231) and the human melanoma cell line (SK-Mel2) were obtained from the American Type Culture Collection (ATCC) and were tested for mycoplasma. These cells were cultured using Iscove’s modified dulbecco’s medium (IMDM) supplemented with 10% fetal bovine serum (FBS) and 10μg/mL gentamycin (complete medium) at 37°C under humidified atmosphere with 5% CO_2_. All cell lines were passaged < 40 times prior to experiments. At the conclusion of this study, all cell lines were authenticated using Illumina sequencing and STARS analysis [28]. All cell studies were conducted using these conditions unless stated otherwise.

All animal studies were conducted in compliance with the Washington University Institutional Animal Care and Use Committee. Female NCI athymic NCr-*nu/nu* (5 – 7 weeks old) were obtained from Charles River and 1.5% isoflurane was used as anesthesia. Xenografts were generated by inoculating the mice with 100 μL of ∼10^7^ cells suspended in PBS, subcutaneously. Tumor sizes reached between 100 - 500 mm^3^ at 4 weeks post inoculation for MDA-MB-468 xenografts and 200 – 500 mm^3^ for MDA-MB-231 at 2 weeks post inoculation. In our hands, MDA-MB-157 cells did not form tumors in this strain of mice and were only used for cell binding studies. PDX models were obtained from the HAMLET Core facility [29].

### Preparation of [^89^Zr]DFO-CR011

CR011 (Celldex Therapeutics) was conjugated via lysine residues to desferrioxamine-*p*-benzyl-isothiocyanate (Macrocyclics, Inc.) metal chelate (DFO-Bz-NCS) with minor modifications from previous studies [23]. The conjugation buffer used in the current study was PBS (pH 9) and the radiolabeling buffer was 10 mM sodium citrate buffer (pH 6.8) to reduce antibody aggregation. Zr-89 was produced in house with an effective specific activity of 145 – 656 mCi/μmol. The highest specific activity of [^89^Zr]DFO-CR011 was achieved at 370 MBq/mg. The ratio of DFO:CR011 was determined using a radioisotopic dilution assay as described previously [30], except that FeCl_3_ was used to compete with Zr-89. For cell-binding and animal studies, a specific activity of 185 MBq/mg was prepared to ensure ≥ 95% radiochemical yield. [^89^Zr]DFO-CR011 was purified by buffer exchanging with 2 mM sodium citrate in saline. Protein aggregation was determined via size exclusion chromatography (Superose 12 10/300 GL) with a flow rate of 0.8 mL/min of 0.1 M sodium phosphate (pH 6.5) containing 0.15 M NaCl buffer. An AKTA fast protein liquid chromatography (FPLC) equipped with radioactivity (Lablogic) and UV (A_280nm_) detectors was used.

### Characterization of the pharmacological properties of [^89^Zr]DFO-CR011 *in vitro*

The specificity of [^89^Zr]DFO-CR011 was evaluated in the TNBC cell lines which express high (MDA-MB-157), moderate (MDA-MB-468), and negative (MDA-MB-231) levels of gpNMB. SKMel-2 melanoma cells were used as the positive control for gpNMB, as this cell line has much higher gpNMB expression levels than most breast cancer cell lines [12]. Each cell line was seeded in 6-well plates in complete medium at 1 × 10^5^cells per well. The medium was changed to 1 mL of starvation medium (1% FBS) and incubated for 24 hrs. All cell studies described in this work were conducted under these conditions. The medium was changed to 1 mL of 50 ng/mL of [^89^Zr]DFO-CR011 (9.25 kBq) in complete media and was incubated for 2 hrs at 37°C. Under blocking conditions, a final concentration of 5 μg/mL of unlabeled CR011 was added to the wells prior to the addition of [^89^Zr]DFO-CR011. Cells were washed with 1 mL of PBS, 3×. Cells were harvested and assayed using an automatic gamma counter (Perkin Elmer), from which the percentage of cell-bound radiotracer was calculated and normalized to the number of cells.

The immunoreactivity of [^89^Zr]DFO-CR011 was determined using a competitive binding assay to compare the ability of unlabeled native CR011 and DFO-CR011 conjugate to compete for binding to gpNMB with [^89^Zr]DFO-CR011. MDA-MB-468 cells (500 μL of 5 × 10^5^ cells/mL) were seeded in 12-well plates exposed to varying concentrations of each competitor, native CR011 or DFO-CR011, ranging from 0 – 25 μg/mL (3-fold dilutions) followed by a final concentration of 0.25 μg/mL of [^89^Zr]DFO-CR011. The reaction was incubated overnight. Cells were washed with PBS, harvested, and assayed in a gamma counter as described above. The percentage of bound radiotracer (B) was normalized to the control reaction where no competitor was added (B_0_). B/B_0_ × 100 was plotted against the log concentration of added competitor. Immunoreactivity was determined to be the ratio between the effective concentrations at 50% (EC_50_) binding between DFO-CR011 and CR011.

The rate of cellular internalization was determined by incubating 250 μL of 50 ng/mL of [^89^Zr]DFO-CR011 with immobilized MDA-MB-468 cells at 37°C from 10 min to 24 hrs. The control reaction was performed at 4°C to show reduced internalization at this temperature. Previous methods were followed for this procedure [31].

### Biodistribution, dosimetry, and PET imaging studies

The pharmacokinetic properties of [^89^Zr]DFO-CR011 were first determined by conducting a biodistribution study in animals bearing MDA-MB-468 tumors. [^89^Zr]DFO-CR011 with a specific activity of 185 MBq/mg (100 μL of 1.85 MBq) was injected via tail vein of the mice. Animals were euthanized at 1, 4, 7, and 12 days post injection (n = 3 per time point). Animals in the blocking study group (n = 3) received 100-fold excess (1 mg) of unlabeled CR011, 1 day prior to injection of the radiolabeled antibody, and were euthanized at 1 and 4 days post injection of [^89^Zr]DFO-CR011. Animals were grouped randomly for these experiments. Organs were harvested, weighed, and assayed in a gamma counter, from which the percentage of injected dose per gram of organ (%ID/g) were calculated. T/B ratios were also calculated to determine the optimal PET time points.

Organ biodistribution data were integrated to generate the organ residence times. These residence times were then scaled to human organ weight and entered in OLINDA/EXM to generate human radiation dose estimates using the adult human female model and using the standard organ weight of ICRP 106 [32, 33]. Heart content was assigned 10% of the blood residence time and all unaccounted residence times was assigned to the remainder of the body.

For small animal PET imaging, 3.7 MBq of [^89^Zr]DFO-CR011 (specific activity = 185 MBq/mg) was injected via tail vein. Static PET images were acquired for 20 min. using an Inveon PET/CT scanner (Siemens, Knoxville, TN) at 4 and 7 days post injection (p.i.). Images were reconstructed using maximum a posteriori algorithm and co-registered with CT images using Inveon Research Workplace Workstation software (Siemens). The [^89^Zr]DFO-CR011 tumor uptake was quantified by drawing regions of interest on the tumor using CT as a guide. The measured radioactivity was decay-corrected to the time of injection of [^89^Zr]DFO-CR011 and the mean standardized uptake values (SUV) were calculated.

### Immunofluorescence

gpNMB was stained using goat anti-human gpNMB (R&D Systems) primary antibody (1:500) at 4°C overnight, then with an anti-goat-Dylight 488 (Novus) secondary antibody (1:2500) at ambient temperature for 1 hr. Slides were counterstained with DAPI mounting solution (Sigma). The high-resolution images of tissue slices were acquired using a digital whole slide scanner (Nanozoomer 2-HT, Hamamatsu. Bridgewater, NJ) using a 20×/0.0.75 lens (Olympus, Center Valley, PA). The images were then quantified using the image analysis module of a digital pathology software Visiomorph (VisioPharm, Broomfield, CO).

### Statistical evaluation

Data was analyzed using GraphPad Prism version 6. A one-way ANOVA was used to analyze statistical significance when comparing samples with one variable, while a 2-way ANOVA was used to analyze samples with 2 variables. P < 0.05 was considered statistically significant.

## SUPPLEMENTARY MATERIALS FIGURES AND TABLE



## Abbreviations

[^89^Zr]DFO-CR011: ^89^Zr-labeled glembatumumab
ADC: antibody drug conjugate
CDX-011: glembatumumab vedotin
CR011: glembatumumab
CT: computed tomography
DFO: desferrioxamine
ELISA: enzyme-linked immunosorbent assay
gpNMB: glycoprotein non-metastatic melanoma B
IHC: immunohistochemistry
PDX: patient derived xenograft
PET: positron emission tomography
SUV: standardized uptake value
TNBC: triple negative breast cancer
Zr-89: zirconium-89
