# Supplementary material for: Preclinical PET imaging of glycoprotein non-metastatic melanoma B in triple negative breast cancer: feasibility of an antibody-based companion diagnostic agent
Source: Oncotarget. 2017 Nov 1;8(61):104303–14. doi: 10.18632/oncotarget.22228 (PMC5732808; doi:10.18632/oncotarget.22228)
Supplement: Supplementary file 1 [file oncotarget-08-104303-s001.pdf]

Preclinical PET imaging of glycoprotein non-metastatic melanoma B in triple negative breast cancer: feasibility of an antibody-based companion diagnostic agent

SUPPLEMENTARY MATERIALS

Flow cytometry

Cells were incubated with 5µg/mL of either a human IgG2 isotype control or CDX-011 mAb for 20 min at room temperature with shaking. After 2 washes, binding was detected with PE-labeled goat anti-human IgG (Fc specific) and samples were read on the flow cytometer.

ELISA

Total gpNMB was quantified in MDA-MB-157, MDA-MB-468, MDA-MB-231, and SK-Mel2 cell lysates. Briefly, cells were lysed in RIPA buffer with protease inhibitor cocktail (Cell Signaling Technology) following supplier instructions. Samples were centrifuged at 14,000 x g for 10 min at 4°C. Supernatant was separated from cell debris and the total protein concentration was determined using BCA assay (Thermo Fisher Scientific). Cell lysates were diluted 100-fold in 1% BSA. Human GPNMB DuoSet ELISA DY2550 (R&D Systems) kit was used to quantify GPNMB based on a standard curve of known concentrations of purified GPNMB (125 – 8000 pg/mL GPNMB).

mRNA microarray

100-500 nanograms(ng) of high quality total RNA from both a tumor biopsy and a reference sample was amplified and either a cy5-CTP(tumor) or cy3-CTP(reference) from Perkin Elmer was incorporated during *in vitro* transcription using Agilent's Low Input Linear Amplification Kit. 825ng of amplified, labeled tumor and reference cRNA were then co-hybridized to an Agilent 4x44K Whole Human Genome microarray (G4112F), washed, and dried according to Agilent's Two-Color Microarray-Based Gene Expression Analysis protocol, version 5.0.1. Processed arrays were then scanned with an Agilent Microarray Scanner (G2505B) and probe data was extracted from the scanned image using Agilent's Feature Extraction software. Feature Extraction data for each patient was then uploaded into the University of North Carolina Microarray Database (UMD), lowess normalized, and log2 ratios (tumor: reference) for each probe were retrieved. Log2 probe data was excluded under any of the following conditions: the probe was not found in either channel; the probes or probe backgrounds were non-uniform outliers; the probe signal was not positive or significant in either channel.

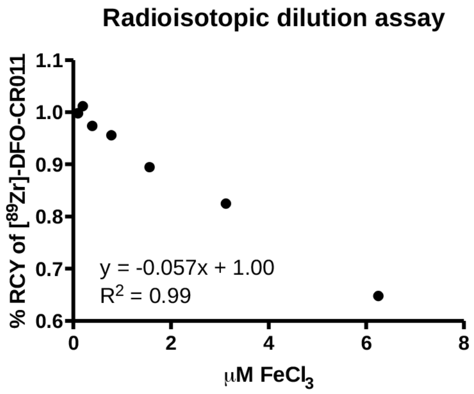

Supplementary Figure 1: The radioisotopic dilution assay determined the DFO:mAb ratio to be 4.

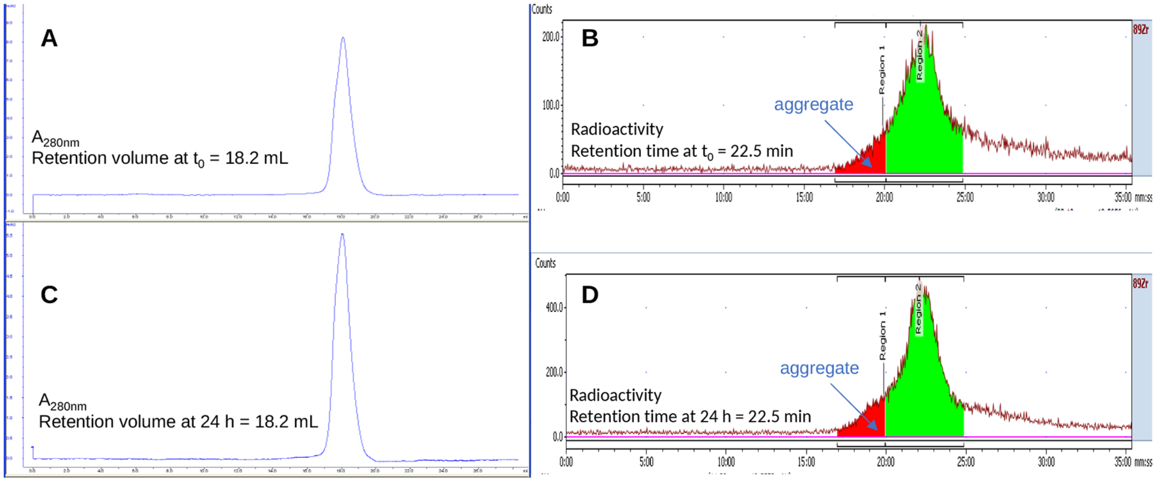

Supplementary Figure 2: Stability study of [^{89}Zr]DFO-CR011 in 2 mM sodium citrate in saline. Chromatograms at t0 are shown in **(A)** (A280) and (**B)** (radioactivity). A_{280} chromatograms show no aggregation at t0 and 24 hrs. Antibody aggregation was identified using the ingrowth of a shoulder peak on the 24 hr time point (17 – 20 min) **(D)**. At t_0, the monomer peak of [^{89}Zr]DFO-CR011 was 93% (B). After 24 hrs, the stability of [^{89}Zr]DFO-CR011 was maintained at 90% monomer (D). The corresponding absorbance at A_{280} are shown in A and **(C)**, respectively.

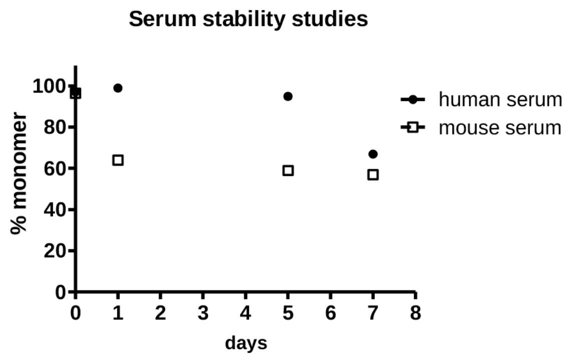

Supplementary Figure 3: Serum stability study of [^{89}Zr]DFO-CR011 up to 7 days in human or mouse serum solutions.

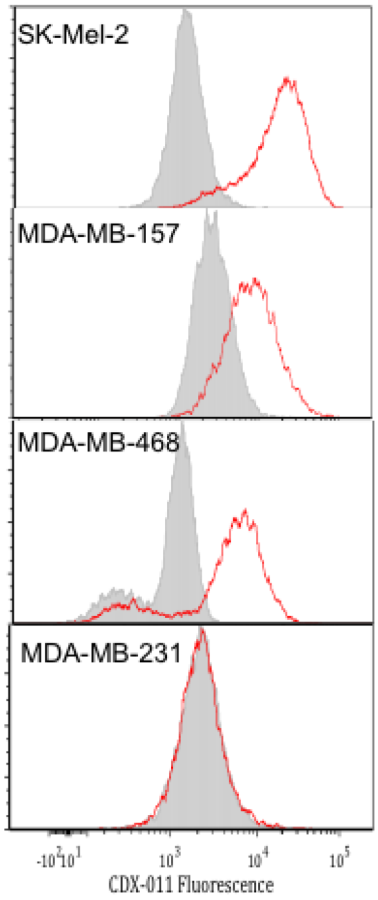

Supplementary Figure 4: Flow cytometry analysis of cell-surface gpNMB in MDA-MB-157, MDA-MB-468, MDA-MB-231, and SK-Mel2, probed using CDX-011 mAb. Human IgG2 mAb was used as an isotype control.

A

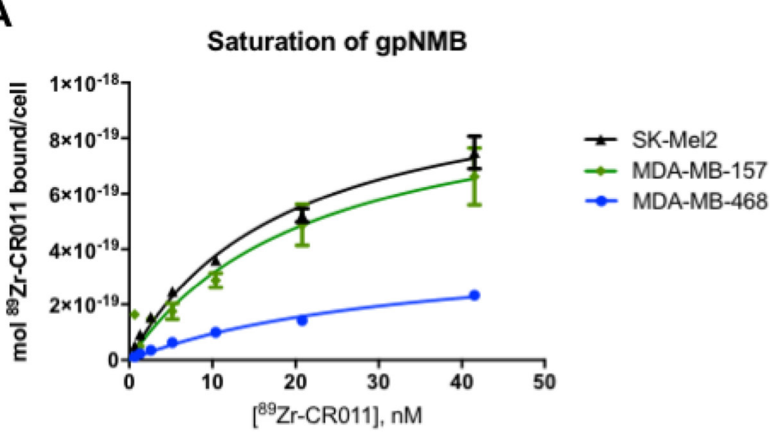

B

	SK-Mel2	MDA-MB-157	MDA-MB-468
Bmax/cell ± SD	1.1 ± 0.02 x 10^{-18} mol	1.0 ± 0.04 x 10^{-18} mol	4.1 ± 0.008 x 10^{-19} mol
# of gpNMB/cell	636,525 ± 11,791	605,211 ± 28,069	246,480 ± 5,017

Supplementary Figure 5: (A) Radioligand saturation assay of [^{89}Zr]DFO-CR011 in SK-Mel2, MDA-MB-157, and MDA-MB-468 cells. Each data point represents triplicate samples with standard deviation. The average binding affinity (Kd) to GPNMB in all three cell lines was determined to be 25 ± 5 nM. **(B)** Summary of the number of gpNMB per cell based on Bmax values calculated from the saturation assay.

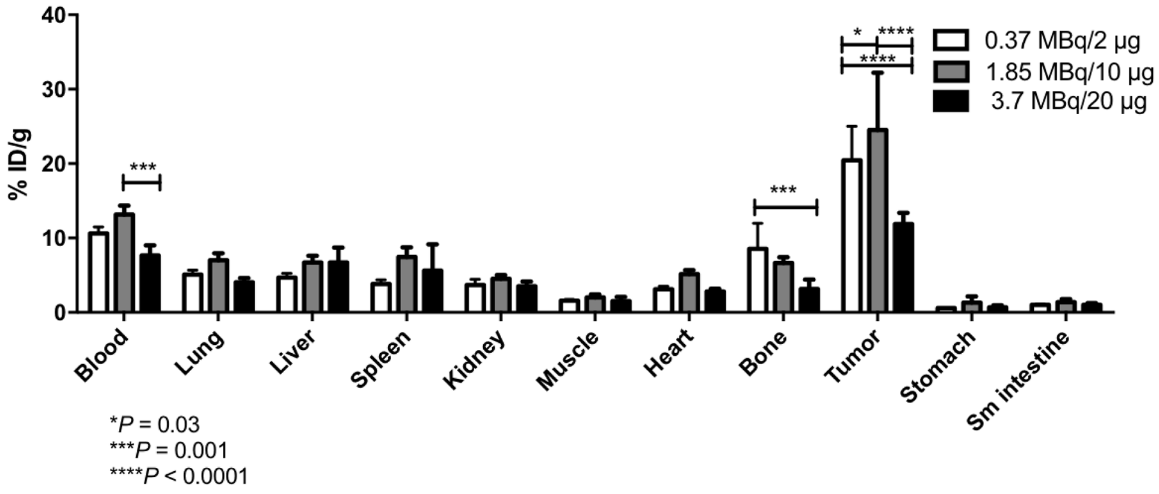

Supplementary Figure 6: Effect of [^{89}Zr]DFO-CR011 dose on its biodistribution in MDA-MB-468 xenografts (n = 3 – 4) at 7 days p. i.

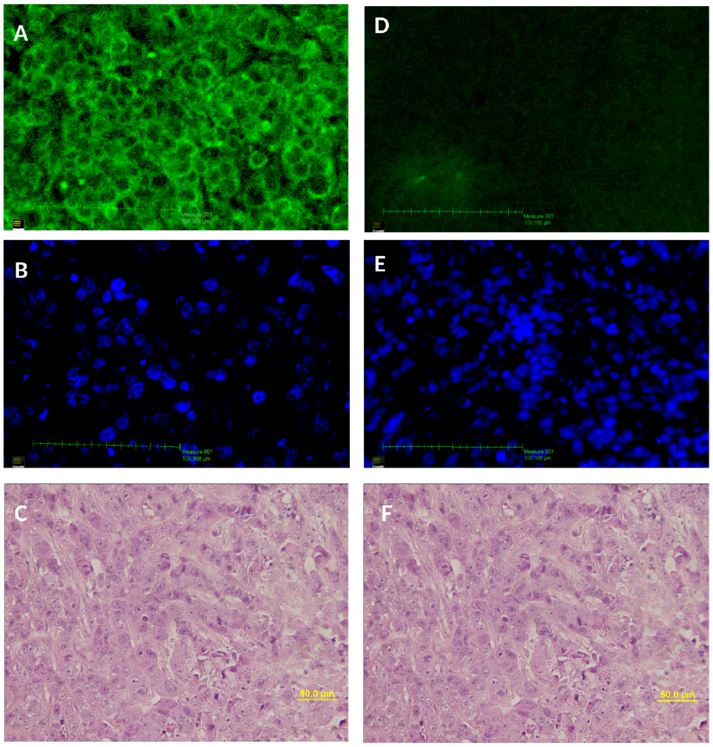

Supplementary Figure 7: Raw data of immunofluorescence staining of gpNMB **(A)**, nuclear staining by DAPI **(B)**, and colorimetric H&E **(C)** in MDA-MB-468. In contrast, MDA-MB-231 images are shown in **(D)** (GPNMB), **(E)** (DAPI), and **(F)** (H&E).

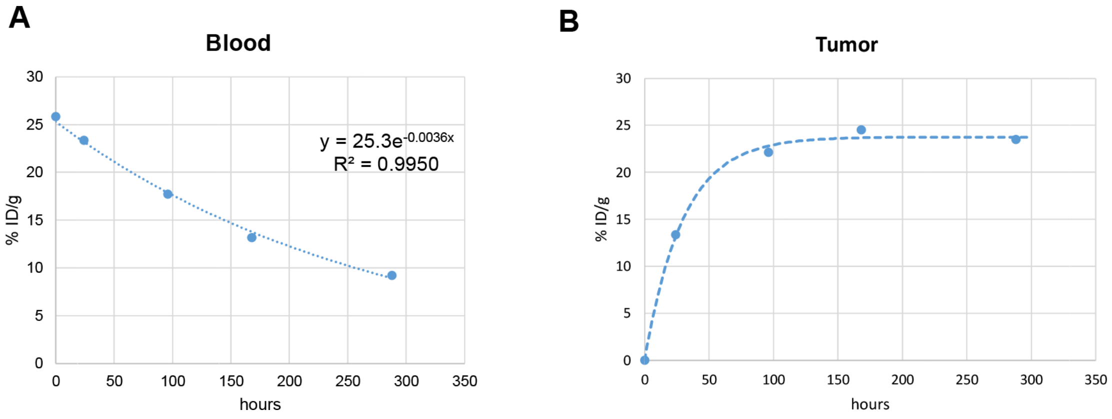

Supplementary Figure 8: Decay-corrected uptake of [^{89}Zr]DFO-CR011 over time. **(A)** In blood, the effective half-life of CR011 was determined to be 8 days. **(B)** In the tumor, the rate of uptake was determined to be 0.034% ID/g/h, in which the half-life of tumor uptake is about 20 hrs.

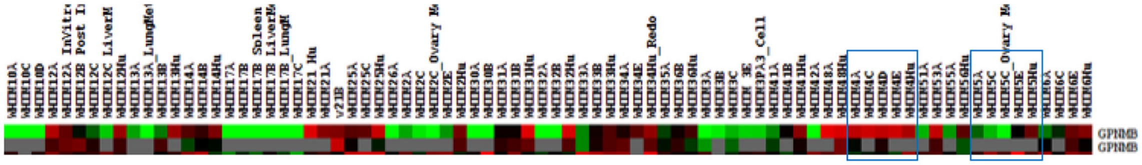

Supplementary Figure 9: gpNMB mRNA microarray analysis of PDX lines and their corresponding human tumors. Out of 27 PDX lines, 10 had upregulated gpNMB expression (red), 9 had downregulated expression(green), 8 lost gpNMB expression from human tissue to mouse passages. Boxed are the PDX models selected for imaging studies: WHIM-4E (upregulated gpNMB) and WHIM-5F (lost expression of gpNMB from human to mouse passages A and C).

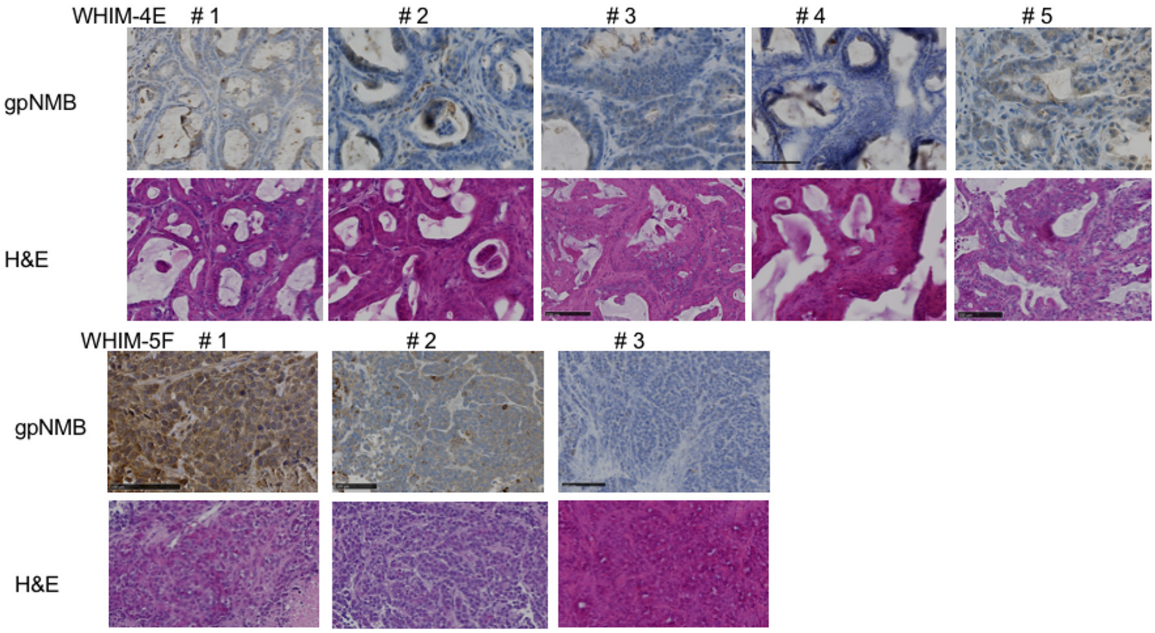

Supplementary Figure 10: Colorimetric IHC analyses for gpNMB protein expression (brown) of WHIM-4E and WHIM-5F mice after PET studies. These results show the heterogeneous expression of gpNMB within the WHIM-5F PDX and the loss of gpNMB expression in this WHIM-4E passage. The corresponding H&E stain for each tissue sample is depicted below its IHC image. Images are shown as 20x magnification.

Supplementary Table 1: Quantification of gpNMB in whole cell lysate using ELISA

Cell line	pg gpNMB/ug total protein
MDA-MB-157	11.1 ± 0.9
MDA-MB-231	0.15 ± 0.05
MDA-MB-468	2.7 ± 0.1
SK-Mel2	6.8 ± 0.3
